# Phosphorus application reduces aluminum toxicity in two *Eucalyptus* clones by increasing its accumulation in roots and decreasing its content in leaves

**DOI:** 10.1371/journal.pone.0190900

**Published:** 2018-01-11

**Authors:** Weichao Teng, Yachao Kang, Wenjuan Hou, Houzhen Hu, Wenji Luo, Jie Wei, Linghui Wang, Boyu Zhang

**Affiliations:** 1 Forestry College, Guangxi University, Nanning, Guangxi, China; 2 Key Laboratory of National Forestry Bureau for Fast-growing Wood Breeding in Central South China, Guangxi University, Nanning, Guangxi, China; 3 Guangxi Colleges and Universities Key Laboratory of Forestry Science and Engineering, Nanning, Guangxi, China; 4 Nanning Dawangtan Reservoir Management, Nanning, Guangxi, China; Estacion Experimental del Zaidin, SPAIN

## Abstract

Under acidic conditions, aluminum (Al) toxicity is an important factor limiting plant productivity; however, the application of phosphorus (P) might alleviate the toxic effects of Al. In this study, seedlings of two vegetatively propagated *Eucalyptus* clones, *E*. *grandis* × *E*. *urophylla* ‘G9’ and *E*. *grandis* × *E*. *urophylla* ‘DH32-29’were subjected to six treatments (two levels of Al stress and three levels of P). Under excessive Al stress, root Al content was higher, whereas shoot and leaf Al contents were lower with P application than those without P application. Further, Al accumulation was higher in the roots, but lower in the shoots and leaves of G9 than in those of DH32-29. The secretion of organic acids was higher under Al stress than under no Al stress. Further, under Al stress, the roots of G9 secreted more organic acids than those of DH32-29. With an increase in P supply, Al-induced secretion of organic acids from roots decreased. Under Al stress, some enzymes, including PEPC, CS, and IDH, played important roles in organic acid biosynthesis and degradation. Thus, our results indicate that P can reduce Al toxicity via the fixation of elemental Al in roots and restriction of its transport to stems and leaves, although P application cannot promote the secretion of organic acid anions. Further, the higher Al-resistance of G9 might be attributed to the higher Al accumulation in and organic acid anion secretion from roots and the lower levels of Al in leaves.

## Introduction

Aluminum (Al) is the most widespread metal and the third most common element in the earth’s crust [1]. In mildly acidic or neutral soils, Al mainly exists in the form of insoluble aluminosilicates and oxides. Several studies have revealed that Al is a beneficial element at low concentrations. Pilon-Smits et al. showed that the beneficial effects of Al in plants are associated with the promotion of growth, activation of antioxidant mechanisms, increases in phosphorus (P) availability, and decreases in Fe toxicity [2]. According to Mitsuru Osaki et al. [3], Al can also be a beneficial element for certain forest species, including *Melaleuca cajuputi* and *Acacia mangium*. However, under acidic conditions, Al toxicity is an important factor limiting plant productivity [4]. Plant species differ in their responses to Al exposure, and numerous studies have revealed that Al stress disrupts a wide range of plant growth and physiological processes under acidic conditions [5]. Inhibition of both root and shoot growth under Al stress has also been commonly reported [6–8]. Although most plants are susceptible to acid-Al stress, some plant species have developed mechanisms that prevent or confer tolerance to Al toxicity, thereby enabling them to survive in environments characterized by excessive Al^3+^. Secretion of organic acids from plant roots has previously been confirmed to be an important mechanism whereby plants relieve Al toxicity [9,10]. Since the first report on the Al-induced secretion of organic acids from wheat (*Triticum aestivum*) roots [11], the results of numerous studies have indicated that many plant species or cultivars, including snapbeans (*Phaseolus vulgaris*) [12], maize (*Zea mays*) [13,14], sickle senna (*Cassia tora*) [15,16], tobacco (*Nicotiana tabacum*) [17], and soybean (*Glycine max*) [18], secrete organic acids from roots under acid-Al stress. In plants subjected to Al stress for prolonged periods, nutrient imbalance can occur, thereby limiting some elements (e.g., calcium, magnesium, nitrogen, phosphorus, and potassium) that are required for plant growth [19,20]. Furthermore, some studies have shown that P deficiency is another important factor that restricts plant growth, particularly in the tropical and subtropical regions, which are characterized by highly weathered acidic soils. Acidic soil contains many chemical compounds, such as ferric oxide and alumina, that fix P in forms that are unavailable to plants [21]. The P use efficiency is very low under Al stress, particularly in acid soils with high levels of toxic Al, because Al-P compounds are insoluble [22]. Even in acidic soils with high concentrations of P, the availability of this nutrient is highly restricted [23]. Previous studies have confirmed that P plays a positive role in alleviating Al toxicity in plants such as wheat [24], snapbeans [12], buckwheat [25], and sorghum[26].

Species of *Eucalyptus* are among the most widely planted trees and have many advantages as a plantation crop. They are used as raw materials in the paper industry, because of the following advantages: strong adaptability, rapid growth, a short rotation period, and the ability to resist drought, and thus provide high economic benefits [27]. However, recent evidence suggests that the productivity of short-rotation plantation forests has reduced [28]. Many factors are known to be associated with the suppression of growth in *Eucalyptus*, acid-induced Al toxicity being one of the most important. Al stress might have detrimental effects on root growth of *Eucalyptus* seedlings, although Al application could promote the secretion of organic acids [29,30]. Yang et al. found that different *Eucalyptus* clones showed different responses to acid-Al stress, which is consistent with the findings of many previous studies [28]. For example, previous studies have shown that *Eucalyptus gummifera* can use insoluble phosphate (aluminum phosphate and iron (III) phosphate) under acidic conditions [31], and that its growth was stimulated in the presence of Al^3+^ [32]. Silva et al. found a slight inhibition of root growth in *E*. *globulus* and *E*. *urophylla*, but only at the highest Al^3+^ level (greater than 500 μM), whereas *E*. *grandis* and *E*. *cloeziana* showed more than 20% reduction in root growth at Al^3+^ levels higher than 256 μM [33]. The resistance to Al toxicity is relatively higher in *E*. *camaldulensis* than in other herbaceous plants [7]. Silva et al. suggested that the higher degree of Al tolerance in *Eucalyptus* species might be associated with the exudation of organic acids [33]. Conversely, some studies have shown that Al treatment inhibits the growth and physiological processes of *Eucalyptus* [30], and a relationship between Al^3+^ tolerance mechanisms and clones (genotypes) has been reported in the literature [33]. However, one of the limitations of these studies is that very little is known regarding the effects of P on the Al-induced-secretion of organic acid anions from the roots of *Eucalyptus* seedlings. Previous studies have indicated that Al plays an important role in inducing the secretion of organic acids, which might chelate Al outside the plasma membrane, thereby preventing its uptake [6,34,35]. Organic acid metabolism involves an array of enzymes, including citrate synthase (CS), phosphoenolpyruvate carboxylase (PEPC), malate dehydrogenase (MDH), NADP-isocitrate dehydrogenase (NADP-ICDH), NADP–malic enzyme (NADP–ME), and aconitase (ACO) [36]. These enzymes are interrelated, function in coordination with each other, and play important roles in organic acid biosynthesis and degradation [35,37]. Koyama et al. suggested that the overexpression of CS in *Arabidopsis thaliana* improves growth in P limited soils because of the enhanced citrate excretion from the roots[38]. Yang et al. suggested that Al- mediated regulation of CS and ACO activities might be responsible for the sufficient citrate efflux, whereas NADP-ICDH, MDH, and PEPC were not directly involved in the Al-induced citrate exudation in the roots of *Cassia tora*[39].

This study aimed to investigate the physiological mechanisms of Al tolerance and Al–P interactions in *Eucalyptus* seedlings. We compared the effects of Al–P interactions on seedling growth; Al and P contents in the roots, stems, and leaves; enzyme activities in the roots; and Al-induced secretion of organic acid anions from the roots of two fast-growing *Eucalyptus* clones under acid-Al treatments.

## Materials and methods

### Study sites

The study was conducted in Nanning, the capital of Guangxi Province, China (108°22ʹE, 22°48ʹN). The area has a subtropical humid climate with an annual average temperature of 21.6°C, ranging from 12.8°C (January) to 28.2°C (July to August). On average, each year, the area has 1827 h of sunshine, a frost-free period of 340 d, relative humidity of 77%, and rainfall of 1304.2 mm. Experiments were conducted in the nursery garden at Guangxi University.

### Experimental design

This study was conducted in a greenhouse under the natural photoperiod occurring at Guangxi University. Two *Eucalyptus* clones, *E*. *grandis* × *E*. *urophylla* ‘G9’ and *E*. *grandis* × *E*. *urophylla* ‘DH32-29’, were used as the experimental materials (labeled G9 and DH32-29, respectively). These two clones have been widely cultivated in Guangxi Province [40]. The experiment was conducted from April to November in 2014. On April 1^st^, we selected healthy and uniformly sized 2-month-old tissue-cultured seedlings. Saplings were grown in porous plastic pots of 250 mm (radius) × 400 mm (height), each containing 50 kg sand. Each pot was watered daily and supplied with 1 L of nutrient solution every week after transplanting. The nutrient solution contained the following macronutrients (in mg/L): CaH_8_N_2_O_10_, 945; KNO_3_, 506; NH_4_NO_3_, 80; KH_2_PO_4_, 136; MgSO_4_, 493; iron salt solution (Formula: FeSO_4_·7H_2_O, 2.78 g; EDTA-Na, 3.73 g; H_2_O, 500 mL), 2.5 mL/L; and microelement solution (Formula (in mg/L): KI, 0.83; H_3_BO_3_, 6.2; MnSO_4_, 22.3; ZnSO_4_, 8.6; Na_2_MoO_4_, 0.25; CuSO_4_, 0.025; CoCl_2_, 0.025), 5 mL/L.

One month after transplanting, treatments were initiated and applied for 20 weeks. The study consisted of six treatments: two levels of Al stress as AlCl_3_·6H_2_O (0 and 5 mM) × three levels of P as KH_2_PO_4_ (0, 50, and 200 μM). The concentrations of Al (0 and 5 mM) used in the present study were determined based on the following information: (1) In a previous field investigation, the soil soluble Al concentration in 5-year-old *Eucalyptus* plantations was approximately 4.4 mM[41]. (2) Ikka et al. showed that 5.0 mM Al stress had a significant effect on the fresh weight, Al content in roots and leaves, and citrate contents and phosphoenolpyruvate carboxylase (PEPC) and aconitase (ACO) activities in the roots of *Eucalyptus camaldulensis*[7]. The concentrations of P (0, 50, and 200 μM) used in the present study were determined based on the following information: (1) A similar previous study on sunflower (*Helianthus annum* L.), subterranean clover (*Trifolium subterraneum* L.), and alfalfa (*Medicago sativa* L.) used P concentrations of 0, 0.8, 8, 80, and 160 μM (as NaH_2_PO_4_-2H_2_O) [42]. (2) Further, another study on two soybean genotypes supplied P at concentrations of 0 and 0.2 mM (as KH_2_PO_4_) [43]. (3) Another study on eight cowpea genotypes supplied P at concentrations of 0 and 100 μM [44]. (4) Moreover, a study on two species of citrus supplied P at concentrations of 0, 50, 100, 250, and 500 μM [45].

Ninety seedlings of each clone (180 seedlings in total) were arranged in a randomized block design and subjected to the six treatments in three blocks, with five pots (one plant per pot) in each treatment in each block (Table 1). Potassium concentration was maintained at a constant level by adding K_2_SO_4_. The pH of the treatment solutions was adjusted to 4.1 or 4.2 by using 2 mol/L HCl or NaOH. Before the treatment was initiated, each pot was supplied with 1 L of nutrient solution every week, and then the pots were supplied with 1 L of nutrient solution every 2 weeks and 1 L of treatment solution every week during the treatment period.

**Table 1:** The randomized block design of different Al-P-clone treatments.

Block 1	Treatment 6	Treatment 10
	Treatment 2	Treatment 4
	Treatment 9	Treatment 1
	Treatment 8	Treatment 7
	Treatment 11	Treatment 3
	Treatment 5	Treatment 12
Block 2	Treatment 1	Treatment 4
	Treatment 11	Treatment 10
	Treatment 12	Treatment 2
	Treatment 6	Treatment 8
	Treatment 7	Treatment 9
	Treatment 3	Treatment 5
Block 3	Treatment 3	Treatment 2
	Treatment 6	Treatment 9
	Treatment 11	Treatment 12
	Treatment 4	Treatment 10
	Treatment 8	Treatment 5
	Treatment 1	Treatment 7

Note: Treatment 1: 0 mM Al, 0 μM P, DH32-29; Treatment 2: 0 mM Al, 50 μM P, DH32-29; Treatment 3: 0 mM Al, 200 μM P, DH32-29; Treatment 4: 5 mM Al, 0 μM P, DH32-29; Treatment 5: 5 mM Al, 50 μM P, DH32-29; Treatment 6: 5 mM Al, 200 μM P, DH32-29; Treatment 7: 0 mM Al, 0 μM P, G9; Treatment 8: 0 mM Al, 50 μM P, G9; Treatment 9: 0 mM Al, 200 μM P, G9; Treatment 10: 5 mM Al, 0 μM P, G9; Treatment 11: 5 mM Al, 50 μM P, G9; Treatment 12: 5 mM Al, 200 μM P, G9. There were five pots (one plant per pot) in each treatment in each block.

### Measurements

#### Measurement of seedling biomass

The biomass of seedlings was measured after harvesting at the end of the study (on September 20). Nine plants per treatment from different pots (one plant per pot) were harvested. The plants were divided into different parts (roots, leaves, and shoots), and then oven-dried for 48 h at 80°C for dry weight (DW) determinations.

#### Analysis of total P and Al in roots, stems, and leaves

After the samples were dried to a constant weight (on September 23), all samples were ground in a mortar to pass through a sieve having pore size of 0.02 mm. Samples were digested with a mixture of HNO_3_:HClO_4_ (5:1, v/v). Mineral (P and Al) contents were then determined using inductively coupled plasma-atomic emission spectroscopy (Agilent Technologies) and the Mo-Sb colorimetric method [46–48].

#### Collection and determination of root exudate organic acids

On September 20, root exudates were collected according to the method of Ryan et al. [49]. Sheared new root tips (5 mm in length) were placed in petri dishes containing 0.5 mM CaCl_2_ (pH 4.1–4.2). After three rinses (each for 20 min), the root tips were transferred to 2-mL centrifuge tubes containing 1 mL control solution in the presence of 0.5 mM AlCl_3_·6H_2_O (pH 4.1–4.2). The tubes were positioned vertically on a shaking platform in the dark for 12 h. After the exudates were filtered through 0.45-μm microporous membranes, the organic acids (malate, citrate, and oxalate) secreted from roots were analyzed using an ICS-5000 chromatography system (Thermo Scientific Dionex ICS-5000; USA).

#### Measurement of enzyme activities in roots

**Preparation of enzyme solution:** On September 20, fresh root (0 to 3 cm; 0.2 g) was placed in a precooled mortar, to which 2 mL grinding buffer (0.2 mol/L Tris-HCl [pH 8.2], 0.6 mol/L sucrose, and 10 mmol/L erythorbic acid) was added, followed by grinding on an ice bath. The preparation was the centrifuged at 4000 × *g* and 4°C for 20 min, and the supernatant was mixed with extraction buffer (0.2 mol/L Tris-HCl [pH 8.2], 10 mmol/L erythorbic acid, and 0.1% Triton X-100) to a constant volume of 5 mL. The resultant supernatant was then treated as follows: (1) 2 mL of supernatant was centrifuged at 15,000 × *g* and 4°C for 15 min, and then the resulting supernatant was mixed with extraction buffer (0.2 mol/L Tris-HCl [pH 8.2], 10 mmol/L erythorbic acid, and 0.1% Triton X-100) to a constant volume of 4 mL to yield the cyt-aconitase (ACO) enzyme solution, next, it was precipitated with the extraction buffer to a constant volume of 2 mL to yield the NAD–isocitrate dehydrogenase (NAD–IDH) enzyme solution. (2) The remaining 3 mL of supernatant was added to 3 mL of extraction buffer for the determination of NAD-malic acid dehydrogenase (NAD-MDH) and NADP-malic enzyme (NADP-ME); 4 mL of this solution was dialyzed against a large volume of extraction buffer overnight at 4°C, and the resulting dialysate was used for the determination of phosphoenolpyruvate carboxylase (PEPC) and citrate synthase (CS).

**Determination of enzyme activities in roots:** PEPC, NAD–MDH, NADP–ME, CS, ACO, and NAD–IDH were extracted and assayed according to Chen and Hirai with some modifications [50,51].

Reactions were performed in a 3-mL volume, and absorbance was determined immediately after the addition of the reaction substrates by using a Lambda 35 UV-Vis ultraviolet spectrophotometer, with continuous scanning for 3 min and measuring absorbance changes of 0.001 per minute for 1 U of enzyme. Enzyme activity is represented in units per gram fresh weight per minute (U/g FW min). The compositions of the reaction systems used for the analysis of the different enzyme activities are as follows:

1. NAD-MDH: 20 × 40 mM Tris-HCl (pH 8.2): 300 μL; 20 × 10 mM KHCO_3_: 150 μL; 20 × 2 mM MgCl_2_: 150 μL; 20 × 0.5 mM GSH: 150 μL; 20 × 0.15 mM NADH: 150 μL; Enzyme liquid: 600 μL; 2 × 2 mM oxaloacetate (initiated reaction): 1500 μL; colorimetric wavelength: 340 nm.
2. NADP-ME: 20 × 40 mM Tris-HCl (pH 7.4): 300 μL; 20 × 0.2 mM MnSO_4_: 150 μL; 20 × 0.17 mM NADP: 150 μL; ultrapure water: 300 μL; enzyme liquid: 600 μL; 2 × 2 mM malate (initiated reaction): 1500 μL; colorimetric wavelength: 340 nm.
3. NAD-IDH: 20 × 40 mM Hepes (pH 8.2): 150 μL; 20 × 0.8 mM NAD: 150 μL; 20 × 0.2 mM MnSO_4_: 150 μL; ultrapure water: 1350 μL; enzyme liquid: 1200 μL; 20 × 2 mM sodium isocitrate (initiated reaction): 300 μL; colorimetric wavelength: 340 nm.
4. PEPC: 20 × 40 mM Tris-HCl (pH 8.5): 150 μL; 20 × 10 mM KHCO_3_: 150 μL; 20 × 2 mM MgCl_2_: 150 μL; 20 × 0.5 mM GSH: 150 μL; 20 × 0.15 mM NADH: 150 μL; ultrapure water: 150 μL; enzyme liquid: 600 μL; 2 × 1 mM PEP (initiated reaction): 1500 μL; colorimetric wavelength: 340 nm.
5. CS: 20 × 40 mM Tris-HCl (pH 9.0): 150 μL; 20 × 0.04 mM DTNB: 150 μL; 20 × 0.04 mM AcCoA: 150 μL; ultrapure water: 1800 μL; enzyme liquid: 600 μL; 20 × 2 mM oxaloacetate (initiated reaction): 150 μL; colorimetric wavelength: 412 nm.
6. ACO (enzyme liquid and the same volume of 2 mM GSH in a 30°C water bath culture for 1 h before the determination of enzyme activity): 20 × 40 mM Tris-HCl (pH 7.5): 150 μL; 20 × 0.1 mM NaCl: 150 μL; ultrapure water: 1080 μL; enzyme liquid: 1200 μL; 20 × 0.2 mM cis-aconitate (initiated reaction): 420 μL; colorimetric wavelength: 340 nm.

### Data analysis

The linear model we used for data analysis is as follows:

$$Y_{ijkl}=\mu +B_{i}+Al_{j}+P_{k}+C_{l}+Al*P_{jk}{}_{}+Al*C_{jl}+P*C_{kl}+Al*P*C_{jkl}+\varepsilon_{ijkl}$$

where *Y* is the individual growth/physiological observation; *μ* is the overall mean; *B*_*i*_ is the effect of the *i*th block (fixed); *Al*_*j*_ is the effect of the *j*th aluminum treatment; *P*_*k*_ is the effect of the *k*th phosphorus treatment; *C*_*l*_ is the effect of the *l*th clone (genotypes); Al*P_*jk*_ is the effect of the statistical interaction between the *j*th aluminum treatment and the *k*th phosphorus treatment; Al*C_*jl*_ is the effect of the statistical interaction between the *j*th aluminum treatment and the *l*th clone; P*C_*kl*_ is the effect of the statistical interaction between the *k*th phosphorus treatment and the *l*th clone; Al*P*C_*jkl*_ is the effect of the statistical interaction among the *j*th aluminum treatment; the *k*th phosphorus treatment and the *l*th clone; and *ε* is the residual error.

All results are expressed as means ± standard deviations. The data were analyzed using analysis of variance (ANOVA) with three factors. When the ANOVA identified differences among groups, multiple comparisons among means were performed using Duncan’s new multiple-range test. The significance level was set at *P ≤ 0*.*05*.

## Results

### Effects of block, Al, P, and clone on seedling growth parameters

The results of variance analysis (Table 2) indicated that individual treatments with Al and P had highly significant effects on the root, stem, and leaf DWs of the two *Eucalyptus* clones. Furthermore, a significant effect of clone was noted on the root, stem, and leaf DWs. Further, significant interactions were noted between Al and P in terms of the biomasses of root, stem, and leaf.

**Table 2:** Variance analysis of the effects of block, Al, P, and clone on the root, stem, and leaf DWs, and root/shoot DW ratio of seedlings.

Source	df	RDW		SDW		LDW		R	
		MS	*P*	MS	*P*	MS	*P*	MS	*P*
B	2	47.95	0.28	49.94	0.43	24.44	0.38	0.00	0.28
Al	1	5077.75	<0.01**	4211.15	<0.01**	8332.65	<0.01**	0.05	<0.01**
P	2	1364.29	<0.01**	811.07	<0.01**	269.26	<0.01**	0.00	0.97
C	1	277.17	0.01 *	438.90	0.01*	119.83	0.04*	0.00	0.69
Al*P	2	151.46	0.03 *	247.10	0.03*	110.68	0.02*	0.00	0.33
P*C	2	150.39	0.03 *	17.78	0.74	15.52	0.53	0.01	0.16
Al*C	1	297.05	<0.01**	909.83	<0.01**	203.63	<0.01**	0.00	0.23
Al*P*C	2	174.78	0.02 *	143.14	0.11	15.49	0.53	0.00	0.80
Error	22	36.32		57.62		23.85		0.00	
Total	35								

Note: df and MS represent degrees of freedom and mean square, respectively. B, Al, P, and C represent the sum of block, aluminum, phosphorus, and clone, respectively. RDW, SDW, LDW, and R, represent root dry weight, stem dry weight, leaf dry weight, and root/shoot ratio, respectively.

* Significant difference (0.01 < *P* < 0.05).

** Highly significant difference (*P* < 0.01)

### Effects of Al–P interactions on seedling growth parameters

**Fig 1:**
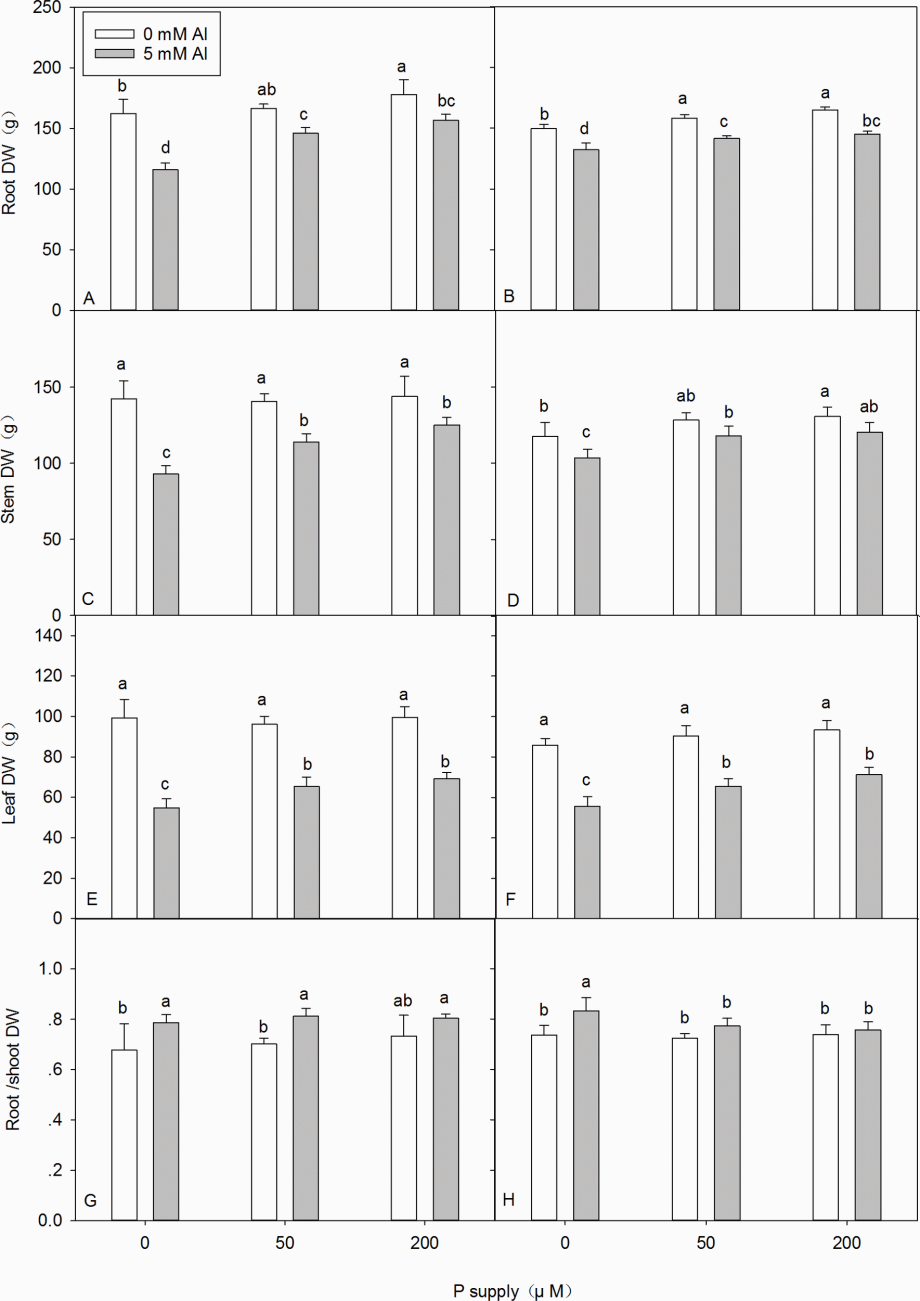
**Root dry weight (DW), shoot DW, and root /shoot DW ratio of DH32-29 (A, C, E, G) and G9 (B, D, F, H) seedlings subjected to different aluminum (Al) and phosphorus (P) treatments.** Bars represent means ± standard deviations (n = 3). Differences among the six treatments were analyzed using 2 (Al) × 3 (P) analysis of variance. Different letters above the bars indicate a significant difference at *P* < 0.05.

The results of this study indicated that the application of P increased, whereas the application of Al decreased, the root, stem, and leaf DW of DH32-29 and G9 (Fig 1A–1F and S1 Table). Regardless of the co-occurrence of Al stress, application of P did not significantly affect the root/shoot DW ratio of DH32-29 (Fig 1G). In contrast, P application resulted in a significant decrease in the root/shoot DW ratio of G9 under Al stress (Fig 1H). However, in the absence of Al stress, P application did not significantly affect the root/shoot DW ratio of G9 (Fig 1H).

### Effects of block, Al, P, and clone on the total Al and P contents in the roots, stems, and leaves

The results of variance analysis (Table 3) indicated highly significant effects of Al, clone and P individual treatments with regard to total Al and P contents in the roots, stems, and leaves of the two *Eucalyptus* clones. Moreover, a significant interaction between Al and P was noted on the total Al and P contents in the roots, stems, and leaves of the two *Eucalyptus* clones, except P content in the roots, which did not change significantly.

**Table 3:** Variance analysis of the effects of block, Al, P, and clone on Al and P contents in the seedlings.

Source	df	RAL		SAL		LAL		RP		SP		LP	
		MS	*P*	MS	*P*	MS	*P*	MS	*P*	MS	*P*	MS	*P*
B	2	0.02	0.18	0.00	0.15	0.00	0.06	0.00	0.18	0.00	0.30	0.01	0.03*
Al	1	10.43	<0.01**	0.21	<0.01**	1.69	<0.01**	0.94	<0.01**	1.08	<0.01**	1.48	<0.01**
P	2	0.14	<0.01**	0.01	<0.01**	0.10	<0.01**	0.19	<0.01**	0.69	<0.01**	0.59	<0.01**
C	1	0.43	<0.01**	0.17	<0.01**	0.07	<0.01**	0.23	<0.01**	1.91	<0.01**	0.39	<0.01**
Al*P	2	0.09	<0.01**	0.01	<0.01**	0.05	<0.01**	0	0.64	0.03	<0.01**	0.01	0.02*
P*C	2	0.01	0.33	0.00	0.03*	0.00	0.09	0	0.96	0.04	<0.01**	0.00	0.28
Al*C	1	0.42	<0.01**	0.02	<0.01**	0.03	<0.01**	0.08	<0.01**	0.00	0.13	0.08	<0.01**
Al*P*C	2	0.05	<0.01**	0	0.70	0.00	0.27	0.01	<0.01**	0.01	<0.01**	0.01	<0.01*
Error	22	0.01		0		0.00		0.00		0.00		0.00	
Total	35												

Note: RAL, SAL, and LAL, and RP, SP, and LP represent aluminum content in the root, stem, and leaf and phosphorus content in the root, stem, and leaf, respectively.

* Significant difference (0.01 < *P* < 0.05).

** Highly significant difference (*P* < 0.01)

### Effects of Al–P interactions on the total Al and P contents in the roots, stems, and leaves

**Fig 2:**
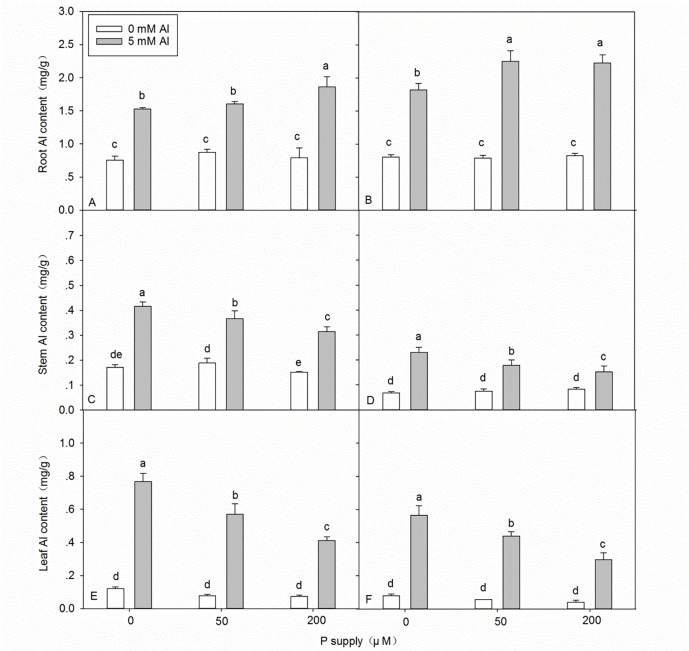
**Aluminum (Al) content in the root, stem, and leaf of DH32-29 (A, C, E) and G9 (B, D, F) seedlings subjected to different Al and P treatments, respectively.** Bars represent means ± standard deviations (n = 3). Differences among the six treatments were analyzed using 2 (Al) × 3 (P) analysis of variance. Different letters above the bars indicate a significant difference at *P* < 0.05.

**Fig 3:**
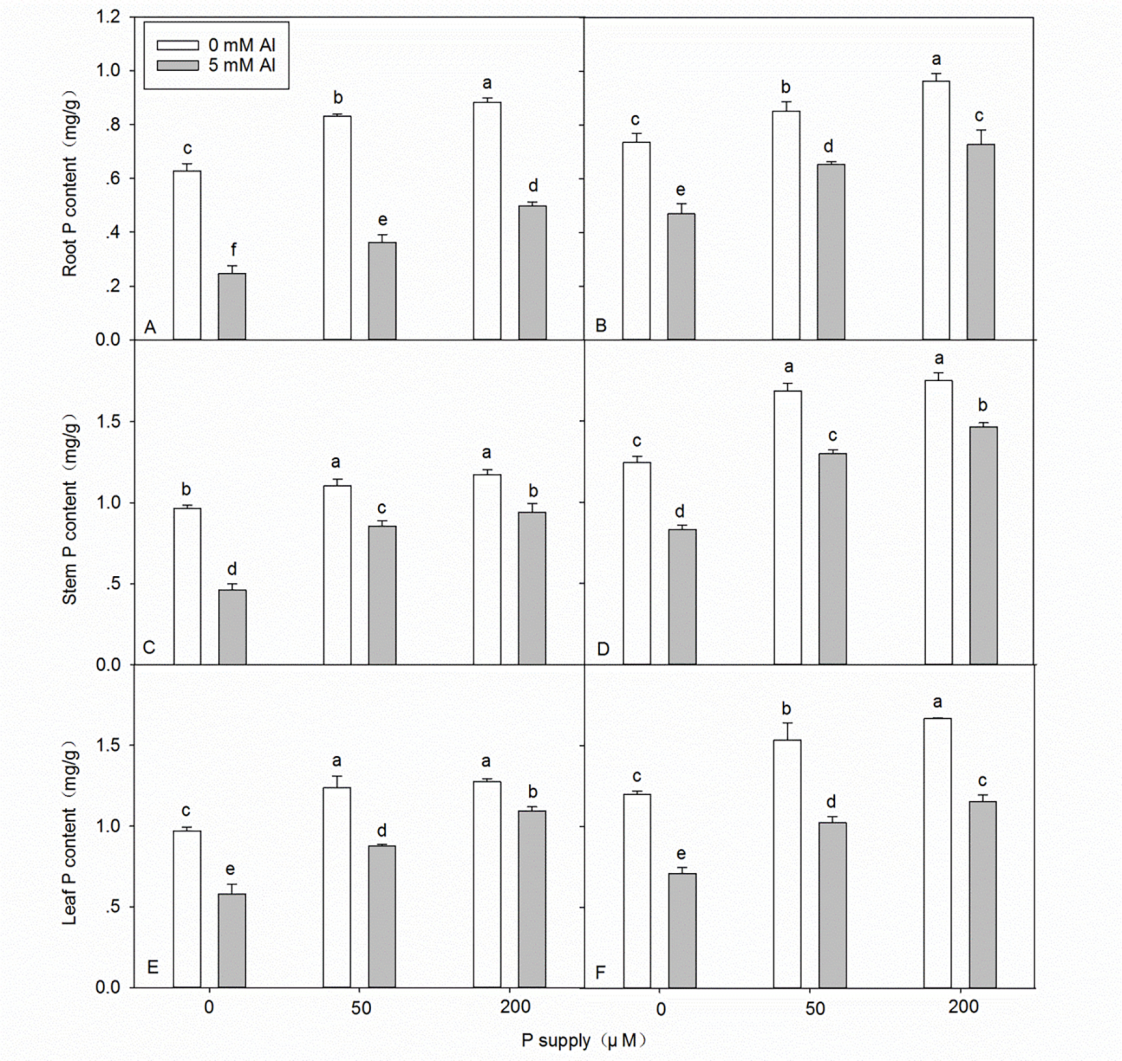
**Phosphorus (P) content in the root, stem, and leaf of DH32-29 (A, C, E) and G9 (B, D, F) seedlings subjected to different Al and P treatments.** Bars represent means ± standard deviations (n = 3). Differences among the six treatments were analyzed using 2 (Al) × 3 (P) analysis of variance. Different letters above the bars indicate a significant difference at *P* < 0.05.

Al stress caused a significant increase in the Al content in seedlings (Fig 2A–2F and S2 Table). In the absence of Al stress, application of P did not significantly affect the Al content in the roots, stems, and leaves of either DH32-29 or G9, except the slight decrease in Al content in the stems of DH32-29 (Fig 2C). Under 5 mM Al stress, after application of P, the Al content in the below-ground plant tissues increased, whereas that in the above-ground plant tissues decreased (Fig 2A–2F). The most interesting finding was that the Al content in the roots of DH32-29 was lower than that in G9, whereas the Al content in the stems and leaves of DH32-29 was higher than that of G9 (Fig 2A–2F and S3 Table). The P contents in the root, stem, and leaf decreased under Al stress (Fig 3A–3F and S2 Table). The P content of seedlings increased with increasing P application, with or without Al stress (Fig 3A–3F), and higher P contents were observed in the roots, stems, and leaves of G9 seedlings than in DH32-29 (Fig 3A–3F and S3 Table).

### Effects of block, Al, P, and clone on Al-induced secretion of organic acids from the roots

The results of variance analysis (Table 4) indicated highly significant effects of Al, clone, and P individual treatments on Al-induced secretion of organic acids from roots. A highly significant interaction between Al and P treatments was noted for Al-induced secretion of two organic acids (malate and citrate) from roots.

**Table 4:** Variance analysis of the effects of block, Al, P, and clone on Al-induced secretion of organic acids from roots.

Source	df	MA		OX		CI	
		MS	*P*	MS	*P*	MS	*P*
B	2	0.03	0.36	0.04	0.20	0.00	0.86
Al	1	28.91	<0.01**	9.39	<0.01**	0.11	<0.01**
P	2	0.90	<0.01**	0.96	<0.01**	0.02	<0.01**
C	1	6.62	<0.01**	6.89	<0.01**	0.24	<0.01**
Al*P	2	2.41	<0.01**	0.02	0.46	0.01	<0.01**
P*C	2	0.01	0.69	0.23	<0.01**	0.01	<0.01**
Al*C	1	0.19	<0.01**	0.07	0.08	0.00	0.22
Al*P*C	2	0.02	0.46	0.14	<0.01**	0.01	<0.01**
Error	22	0.02		0.02		0.00	
Total	35						

Note: MA, OX, and CI represent Al-induced secretion of malate, oxalate, and citrate from roots, respectively.

* Significant difference (0.01 < *P* < 0.05).

** Highly significant difference (*P* < 0.01)

### Effects of Al–P interactions on Al-induced secretion of organic acids from the roots

**Fig 4:**
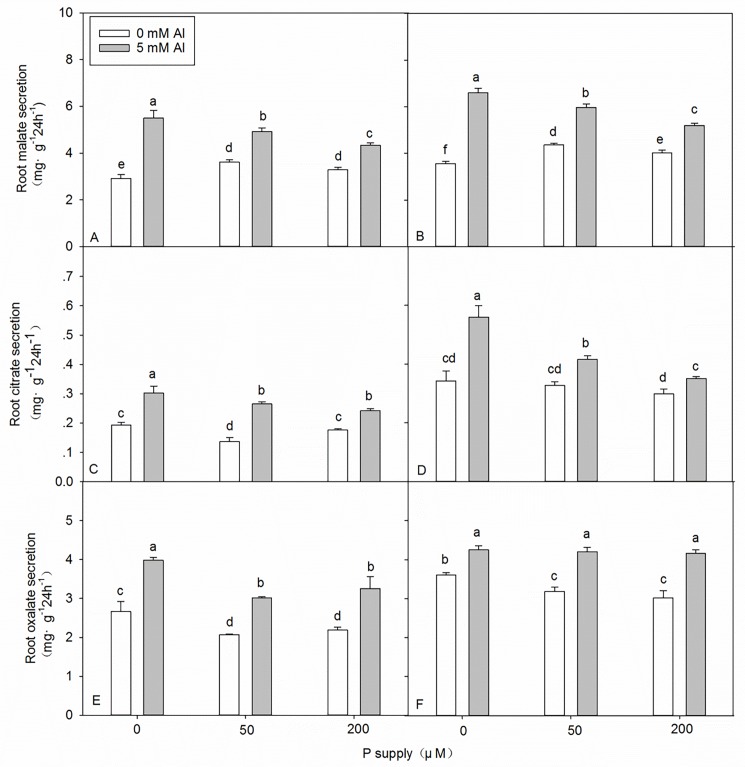
**Secretion of malate, oxalate, and citrate from the roots of DH32-29 (A, C, E) and G9 (B, D, F) seedlings treated with different levels of Al and P.** Bars represent means ± standard deviations (n = 3). Differences among the six treatments were analyzed using 2 (Al) × 3 (P) analysis of variance. Different letters above the bars indicate a significant difference at *P* < 0.05.

In the absence of Al stress, the Al-induced secretion of malate from DH32-29 and G9 roots was significantly higher after the application of 50 and 200 μM P than after the application of 0 μM P (Fig 4A and 4B). In contrast, under the same conditions, the secretion of oxalate from DH32-29 and G9 roots was significantly lower after the application of 50 and 200 μM P than after the application of 0 μM P (Fig 4E and 4F). The secretion of citrate from G9 roots did not change significantly with increasing P supply, whereas that from DH32-29 roots was significantly lower after the application of 50 μM P than after the application of 0 and 200 μM P (Fig 4C and 4D). Under Al stress, the Al-induced secretion of organic acids from DH32-29 and G9 roots decreased significantly with increasing P supply, except the secretion of oxalate from the roots of G9, which did not change significantly (Fig 4A–4F).

### Effects of block, Al, P, and clone on the enzyme activities in the roots

The results of variance analysis (Table 5) indicated highly significant effects of Al, clone, and P individual treatments on enzyme activities in roots, except MDH activity in roots, which was not sensitive to P treatment. In roots, a highly significant interaction between Aland P treatments was noted for the activities of four enzymes, i.e., PEPC, ME, IDH, and ACO, and a significant interaction was noted for two enzymes, i.e., MDH and CS.

**Table 5:** Variance analysis of the effects of block, Al, P, and clone on enzyme activities in roots.

Source	df	PE		ME		MD		CS		ID		AC	
		MS	*P*	MS	*P*	MS	*P*	MS	*P*	MS	*P*	MS	*P*
B	2	415.87	0.40	0.05	0.99	11.43	0.18	587.11	0.09	39.50	0.16	15.24	0.63
Al	1	194882.52	<0.01**	1528.55	<0.01**	13738.58	<0.01**	16412.17	<0.01**	11065.29	<0.01**	60583.44	<0.01**
P	2	13042.43	<0.01**	377.90	<0.01**	11.31	0.186	3662.20	<0.01**	311.14	<0.01**	2359.64	<0.01**
C	1	221505.72	<0.01**	3917.51	<0.01**	10795.56	<0.01**	74165.26	<0.01**	1157.25	<0.01**	13137.36	<0.01**
Al*P	2	19834.39	<0.01**	469.50	<0.01**	33.16	0.01*	1274.38	0.01*	1232.34	<0.01**	1200.03	<0.0**
P*C	2	2760.22	<0.01**	34.54	<0.01**	267.91	<0.01**	488.30	0.13	56.93	0.08	313.39	<0.01**
Al*C	1	6415.21	<0.01**	7.09	0.27	10911.54	<0.01**	1259.30	0.03*	333.12	<0.01**	3246.53	<0.01**
Al*P*C	2	438.89	0.38	62.58	<0.01**	103.96	<0.01**	1589166.00	<0.01**	56.72	0.08	147.30	0.02*
Error	22	435.35		5.53		6.21		221.68		19.85		31.99	
Total	35												

Note: PE, ME, MD, CS, ID, and AC represent the activity of PEPC, NADP-ME, NAD-MDH, CS, NAD-IDH, and Cyt-ACO in roots, respectively.

* Significant difference (0.01 < *P* < 0.05).

** Highly significant difference (*P* < 0.01)

### Effects of Al–P interactions on enzyme activities in roots

**Fig 5:**
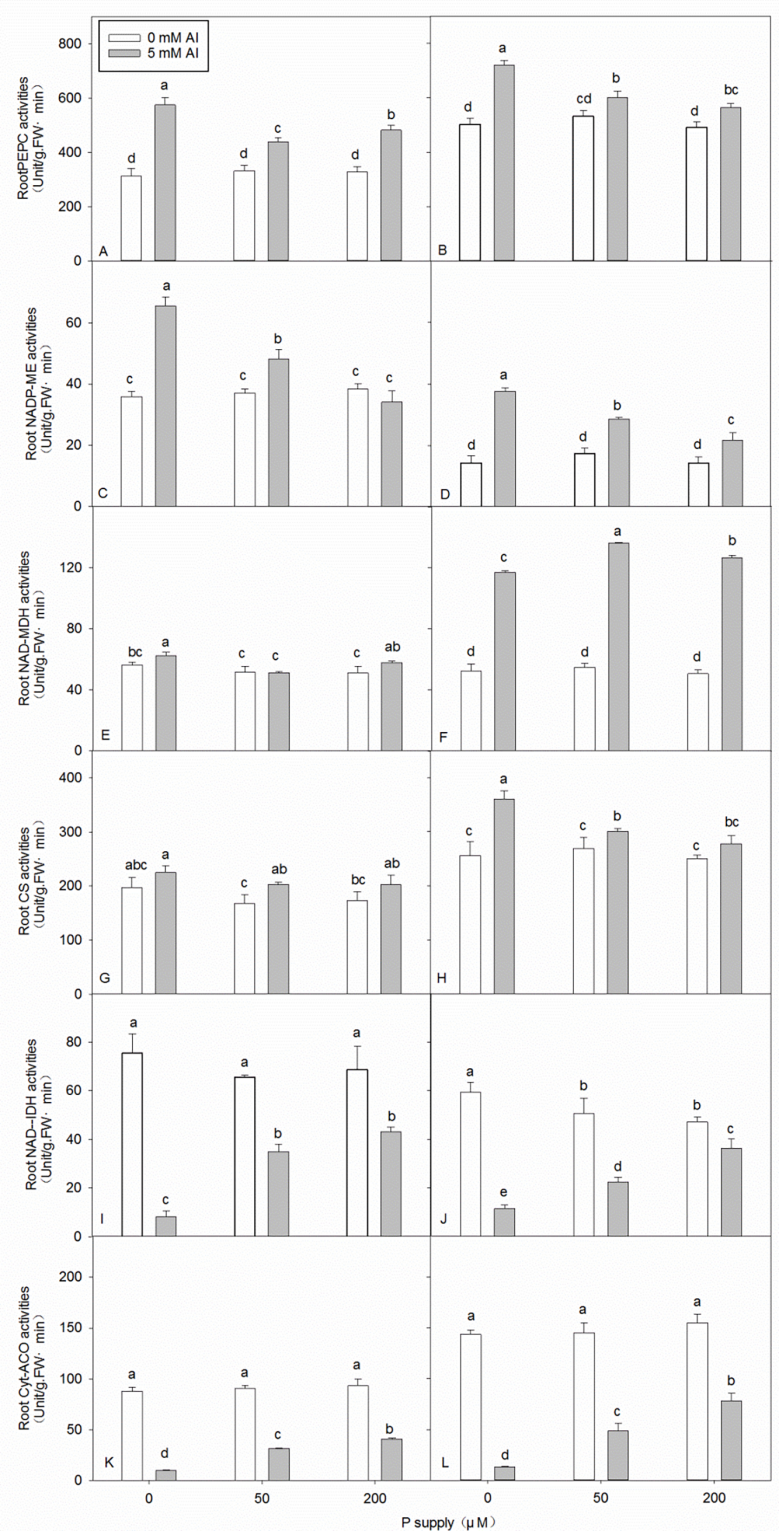
**Root enzyme activities in DH32-29 (A, C, E, G, I, K) and G9 (B, D, F, H, J, L) seedlings treated with different levels of Al and P.** Bars represent means ± standard deviations (n = 3). Differences among the six treatments were analyzed using 2 (Al) × 3 (P) analysis of variance. Different letters above the bars indicate a significant difference at *P* < 0.05.

In neither clone, the PEPC activity was significantly affected by P supply without Al stress, whereas it was significantly lower after the application of 50 and 200 μM P than after the application of 0 μM P with Al stress (Fig 5A and 5B). In both the clones, Al increased PEPC activity (Fig 5A and 5B and S6 Table).

The change in the trend of NADP–ME activity was similar to that of PEPC activity. NADP–ME activity did not change significantly regardless of P supply in the absence of Al stress, but decreased significantly with increasing P supply under Al stress (Fig 5C and 5D).

In both the clones, NAD–MDH activity showed no significant change irrespective of P supply in the absence of Al stress (Fig 5E and 5F). Furthermore, under Al stress, in DH32-29, NAD–MDH decreased significantly with an increase in P supply from 0 to 50 μM, but increased slightly after the application of 200 μM P (Fig 5E). In contrast, NAD–MDH in G9 showed the reverse trend, with its activity being higher after the application of 50 μM P than after the application of either 0 or 200 μM P (Fig 5F). In both the clones, Al increased NAD–MDH activity, except the application of 50 μM P of DH32-29, which did not change significantly (Fig 5E and 5F).

In both the clones, CS activity did not change significantly with or without P supply in the absence of Al stress (Fig 5G and 5H). Under Al stress, CS activity in DH32-29 did not change significantly with increasing P supply, whereas decreased significantly in G9 (Fig 5G and 5H). In G9, Al increased CS activity in 0 or 50 μM P application; in DH32-29, Al increased CS activity only in 50 μM P application (Fig 5G and 5H).

The change in IDH activity showed a different trend to that of the aforementioned enzymes (Fig 5I and 5J); Al stress significantly inhibited IDH activity (S6 Table). In DH32-29, IDH activity was not significantly affected by P supply without Al stress; however, with increasing P supply, the NAD-IDH activity of G9 roots decreased significantly in the absence of Al stress, whereas increased significantly under Al stress (Fig 5I and 5J).

The change trend in ACO activity was similar to that of IDH activity. In both the clones, ACO activity was not significantly affected by P supply without Al stress. Furthermore, ACO activity increased significantly with increasing P supply under Al stress (Fig 5K and 5L). Like for NAD–IDH, Al decreased ACO activity in both the clones (Fig 5K and 5L and S6 Table).

## Discussion

### Application of Al and P affects the dry biomass of *Eucalyptus* seedlings

Several studies have indicated that different *Eucalyptus* species have different degrees of resistance to Al toxicity. Moreover, differences in tolerance to Al toxicity between the *Eucalyptus* clones were detected by Yang et al. [28]. In this study, Al stress was found to have significant toxic effects on the root, stem, and leaf biomass growth of *Eucalyptus* seedlings (Fig 1A–1F and S1 Table). Al stress resulted in an increase in the root/shoot DW ratio (Fig 1G and 1H and S1 Table). The patterns of root/shoot biomass allometry indicated that biomass growth was greater in *Eucalyptus* seedlings roots than in the stems and leaves under Al stress, and *Eucalyptus* seedlings might have changed their biomass allocation patterns or structures in response to environmental Al stress. These finding were similar to those in cowpea [44] and citrus [52]. Furthermore, our data indicated that the application of P promotes the growth of *Eucalyptus* seedlings with and without Al stress (Fig 1A–1F), and that, with the application of 200 μM phosphorus, no significant difference in the root/shoot DW ratio was noted between plants with or without Al stress in both the clones (Fig 1G and 1H). This indicates that the application of P might play a positive role in enhancing the ability of plants to resist Al stress.

### Application of Al and P affects their concentrations in *Eucalyptus* seedlings

In our study, the P concentration in both *Eucalyptus* clones was significantly reduced by Al stress (Fig 3A–3F and S2 Table). Conversely, Moreno-Alvarado et al. found a synergic effect of Al on P [53]. This difference could be related to the fact that Mexican rice cultivars can absorb higher amounts of H^+^ as well as prevent cytoplasmic acidification [53,54]. The discrepancy between our findings and those of Moreno-Alvarado et al. might also be attributed to several factors such as the use of different species (*Eucalyptus* seedlings vs. rice cultivars); different nutrient solutions (Hoagland nutrient solution with some modifications vs. Yoshida nutrient solution); different cultivation modes (sand culture vs. hydroponic assay); different Al treatment levels (5 mM vs. 200 μM); and different age of plants before exposure to Al (3 months vs. 24 days). Previous studies have shown that plant resistance to Al toxicity might be mediated by not only a decrease in Al uptake but also a relatively lower proportion of Al being distributed from roots to shoots and leaves [55,56]. In our study, under Al stress, application of P increased Al content in the below-ground plant tissues, but decreased its content in the above-ground plant tissues (Fig 2A–2F). According to the previous views, like Zhang and Matsumoto (2005), they found Al-P precipitates can accumulate in the root cell wall, and these precipitates might be helpful by retarding the uptake of Al into the cytosol, therefore, they are generally considered non-toxic to plants [25]. In this experiment, non-toxic Al–P compounds might be increased in the root tissues of *Eucalyptus* seedlings with increasing P supply under Al stress, resulting in decreased transport of Al to stems and leaves. This could be a strategy that enables plants to tolerate Al toxicity. Several studies in other species have revealed similar results [25,45]. Interestingly, in the present study, we found that the Al content in roots was lower in DH32-29 than in G9, whereas that in stems and leaves was higher in DH32-29 than in G9 (Fig 2A–2F and S3 Table). Furthermore, we found that the P content in roots, stems, and leaves was lower in DH32-29 than in G9 (Fig 3A–3F and S3 Table). This difference suggests that, unlike DH32-29, G9 might have developed a more efficient mechanism to uptake P and restrict Al transport to the stems and leaves. Based on the findings of the present study, we propose the following two mechanisms to explain the enhancement of Al resistance in the two clones of *Eucalyptus* following the application of P: (1) accumulation of Al by P present in the roots and restriction of Al transport to the stems and leaves; and (2) an increase in P concentration in the roots, stems, and leaves that alleviates elemental deficiency.

### The application of P affects the exudation of organic acids by roots under Al stress

The Al-induced exudation of organic acids from plant roots has been proposed to be an important mechanism for Al tolerance [39]. The results of our study indicate that Al stress promoted the exudation of organic acids (Fig 4A–4F and S4 Table). However, under Al stress, Al-induced secretion of malate, oxalate, and citrate from DH32-29 and G9 roots did not increase with increasing P supply (Fig 4A–4F), indicating that Al resistance might not be explained by the fact that the application of P increased the exudation of organic acids. This response implies that the decreased exudation of organic acids with increasing P application could be because of the amelioration of Al toxicity in *Eucalyptus* clones. Studies such as those conducted by Gaume et al. [57], Liang [58] and Sun [59] have obtained similar results. In addition, different genotypes show different extents of organic acid exudation [57]. We also found that, unlike DH32-29, G9 had developed more efficient mechanisms to secrete organic acids from roots (Fig 4A–4F and S5 Table). This might be one of the reasons why Al resistance of G9 was stronger than that of DH32-29. Previous studies have shown that the expression level of *NAC* transcription factor genes might be regulated by Al [53]. Hussey et al. identified 189 nonredundant *NAC* domain proteins in the *E*. *grandi*s genome, one of the largest *NAC* domain families known [53,60]. Further studies are required to elucidate the differences in the expression of *NAC* gene family members in the two clones of *E*. *grandi*.

The metabolism of organic acids in roots is an important factor regulating the secretion of organic acids and, under Al stress, the secretion of organic acids might be associated with the changes in enzyme activity. In our study, the Al-induced secretion of malate and oxalic under Al pretreatment (Fig 4A, 4B, 4E and 4F and S4 Table) was higher than that with no Al pretreatment, which could generally be attributed to increased biosynthesis, as indicated by the increased activity of PEPC in *Eucalyptus* seedlings (Fig 5A and 5B and S6 Table). Furthermore, the Al-induced secretion of citrate under Al pretreatment (Fig 4C and 4D and S4 Table) was higher than that under no Al pretreatment, which could generally be attributed to the increased biosynthesis, as indicated by the increased activities of PEPC and CS in *Eucalyptus* seedlings (Fig 5A, 5B, 5G and 5H and S6 Table) and decreased degradation, as indicated by the decreased IDH activity in both the clones (Fig 5I and 5J and S6 Table). Under Al pretreatment, the Al-induced secretion of the three types of organic acids decreased with an increase in applied P (Fig 4A–4E), with the exception of oxalic acid (Fig 4F); this could generally be attributed to the decreased biosynthesis, as indicated by the decreased activities of PEPC and CS in *Eucalyptus* seedlings (Fig 5A, 5B, 5G and 5H) and increased degradation, as indicated by the increased IDH activity in both the clones (Fig 5I and 5J). The activities of PEPC and CS in G9 were higher than those in DH32-29, whereas that of IDH in G9 was lower than that in DH32-29 (Fig 5A, 5B, 5G, 5H, 5I and 5J and S7 Table). These findings, at least in part, confirm that the secretion of organic acids is greater in G9 than in DH32-29.

Two different types of physiological mechanisms of Al tolerance are known: those that operate to exclude Al from the root apex and those that allow plants to tolerate Al accumulation in the root or shoot symplasm [10]. The first mechanism includes those employing root Al exclusion based on Al-activated organic acid exudation from the root apex [10]. The latter type of Al tolerance mechanism encompasses those relying on the conversion of accumulated Al into non-toxic Al-chelating compounds [60]. Our experimental data suggest that Al tolerance in the two *Eucalyptus* clones is a complex trait.

In conclusion, Al stress inhibited growth and decreased the uptake of P in the two *Eucalyptus* clones. P application can reduce Al toxicity by facilitating the fixation of elemental Al in the roots and restricting Al transport to the stems and leaves; however, application of P does not promote the secretion of organic acid anions. The higher Al resistance of G9 might be attributed to the higher Al accumulation and organic acid anion secretion in the roots and the lower Al content in the leaves.

## Supporting information

S1 Table Duncan’s multiple range test with or without Al stress for four growth indexes of seedlings. Note: The abbreviations RDW, SDW, LDW, and R represent root dry weight, stem dry weight, leaf dry weight, and root/shoot ratio, respectively. Differences between the two Al levels were analyzed by ANOVA. Different letters in each row indicate significant differences (Duncan’s test; P ≤ 0.05).
(DOCX)

S2 Table Duncan’s multiple range test with or without Al stress for Al and P contents in seedlings. Note: The abbreviations RAL, SAL, and LAL and RP, SP, and LP represent aluminum content in root, stem, and leaf and phosphorus content in root, stem, and leaf, respectively. Differences between the two Al levels were analyzed by ANOVA. Different letters in each row indicate significant differences (Duncan’s test; P ≤ 0.05).
(DOCX)

S3 Table Duncan’s multiple range test in different clones for Al and P contents in seedlings. Note: Differences between the two clones were analyzed by ANOVA. Different letters in each row indicate significant differences (Duncan’s test; P ≤ 0.05).
(DOCX)

S4 Table Duncan’s multiple range test with or without Al stress for Al-induced secretion of organic acids from roots. Note: The abbreviations MA, OX, and CI represent Al-induced secretion of malate, oxalate, and citrate from roots, respectively. Differences between the two Al levels were analyzed by ANOVA. Different letters in each row indicate significant differences (Duncan’s test; P ≤ 0.05).
(DOCX)

S5 Table Duncan’s multiple range test in different clones for Al-induced secretion of organic acids from roots. Note: Differences between the two clones were analyzed by ANOVA. Different letters in each row indicate significant differences (Duncan’s test; P ≤ 0.05).
(DOCX)

S6 Table Duncan’s multiple range test with or without Al stress for enzyme activities in roots. Note: The abbreviations PE, ME, MD, CS, ID, and AC represent the activities of PEPC, NADP-ME, NAD-MDH, CS, NAD-IDH, and Cyt-ACO in roots, respectively. Differences between the two Al levels were analyzed by ANOVA. Different letters in each row indicate significant differences (Duncan’s test; P ≤ 0.05).
(DOCX)

S7 Table Duncan’s multiple range test in different clones for enzyme activities in roots. Note: Differences between the two clones were analyzed by ANOVA. Different letters in each row indicate significant differences (Duncan’s test; P ≤ 0.05).
(DOCX)
