# Supplementary material for: Phosphorus application reduces aluminum toxicity in two Eucalyptus clones by increasing its accumulation in roots and decreasing its content in leaves
Source: PLoS One. 2018 Jan 11;13(1):e0190900. doi: 10.1371/journal.pone.0190900 (PMC5764327; doi:10.1371/journal.pone.0190900)
Supplement: S1 Table — Note: The abbreviations RDW, SDW, LDW, and R represent root dry weight, stem dry weight, leaf dry weight, and root/shoot ratio, respectively. Differences between the two Al levels were analyzed by ANOVA. Different letters in each row indicate significant differences (Duncan’s test; P ≤ 0.05). (DOCX) [file pone.0190900.s001.docx]

S1 Table. Duncan’s multiple range test with or without Al stress for four growth indexes of seedlings

| Al (mM) | RDW | SDW | LDW | R |
| --- | --- | --- | --- | --- |
| 0 | 163.29 ± 10.79 a | 133.96 ± 12.33 a | 94.08 ± 6.85 a | 0.72 ± 0.06 b |
| 5 | 139.54 ± 13.66 b | 112.33 ± 12.30 b | 63.66 ± 7.36 b | 0.79 ± 0.04 a |

Note: The abbreviations RDW, SDW, LDW, and R represent root dry weight, stem dry weight, leaf dry weight, and root/shoot ratio, respectively. Differences between the two Al levels were analyzed by ANOVA. Different letters in each row indicate significant differences (Duncan’s test; P ≤ 0.05).
