# Supplementary material for: Phosphorus application reduces aluminum toxicity in two Eucalyptus clones by increasing its accumulation in roots and decreasing its content in leaves
Source: PLoS One. 2018 Jan 11;13(1):e0190900. doi: 10.1371/journal.pone.0190900 (PMC5764327; doi:10.1371/journal.pone.0190900)
Supplement: S2 Table — Note: The abbreviations RAL, SAL, and LAL and RP, SP, and LP represent aluminum content in root, stem, and leaf and phosphorus content in root, stem, and leaf, respectively. Differences between the two Al levels were analyzed by ANOVA. Different letters in each row indicate significant differences (Duncan’s test; P ≤ 0.05). (DOCX) [file pone.0190900.s002.docx]

S2 Table. Duncan’s multiple range test with or without Al stress for Al and P contents in seedlings

| Al (mM) | RAL | SAL | LAL | RP | SP | LP |
| --- | --- | --- | --- | --- | --- | --- |
| 0 | 0.81 ± 0.07 b | 0.12 ± 0.05 b | 0.08 ± 0.03 b | 0.82 ± 0.11 a | 1.32 ± 0.30 a | 1.31 ± 0.24 a |
| 5 | 1.88 ± 0.30 a | 0.28 ± 0.10 a | 0.51 ± 0.16 a | 0.49 ± 0.17 b | 0.98 ± 0.34 b | 0.91 ± 0.22 b |

Note: The abbreviations RAL, SAL, and LAL and RP, SP, and LP represent aluminum content in root, stem, and leaf and phosphorus content in root, stem, and leaf, respectively. Differences between the two Al levels were analyzed by ANOVA. Different letters in each row indicate significant differences (Duncan’s test; P ≤ 0.05).
