# Supplementary material for: Phosphorus application reduces aluminum toxicity in two Eucalyptus clones by increasing its accumulation in roots and decreasing its content in leaves
Source: PLoS One. 2018 Jan 11;13(1):e0190900. doi: 10.1371/journal.pone.0190900 (PMC5764327; doi:10.1371/journal.pone.0190900)
Supplement: S3 Table — Note: Differences between the two clones were analyzed by ANOVA. Different letters in each row indicate significant differences (Duncan’s test; P ≤ 0.05). (DOCX) [file pone.0190900.s003.docx]

S3 Table. Duncan’s multiple range test in different clones for Al and P contents in seedlings

| Clone | RAL | SAL | LAL | RP | SP | LP |
| --- | --- | --- | --- | --- | --- | --- |
| DH 32-29 | 1.24 ± 0.46 b | 0.27 ± 0.11 a | 0.34 ± 0.28 a | 0.57 ± 0.24 b | 0.92±0.24 b | 1.01±0.24 b |
| G9 | 1.45 ± 0.69 a | 0.13 ± 0.06 b | 0.25 ± 0.21 b | 0.73 ± 0.16 a | 1.38±0.32 a | 1.21±0.33 a |

Note: Differences between the two clones were analyzed by ANOVA. Different letters in each row indicate significant differences (Duncan’s test; P ≤ 0.05).
