# Supplementary material for: Phosphorus application reduces aluminum toxicity in two Eucalyptus clones by increasing its accumulation in roots and decreasing its content in leaves
Source: PLoS One. 2018 Jan 11;13(1):e0190900. doi: 10.1371/journal.pone.0190900 (PMC5764327; doi:10.1371/journal.pone.0190900)
Supplement: S4 Table — Note: The abbreviations MA, OX, and CI represent Al-induced secretion of malate, oxalate, and citrate from roots, respectively. Differences between the two Al levels were analyzed by ANOVA. Different letters in each row indicate significant differences (Duncan’s test; P ≤ 0.05). (DOCX) [file pone.0190900.s004.docx]

S4 Table. Duncan’s multiple range test with or without Al stress for Al-induced secretion of organic acids from roots

| Al (mM) | MA | OX | CI |
| --- | --- | --- | --- |
| 0 | 3.63 ± 0.49 b | 2.79 ± 0.57 b | 0.25 ± 0.08 b |
| 5 | 5.42 ± 0.77 a | 3.81 ± 0.52 a | 0.36 ± 0.11 a |

Note: The abbreviations MA, OX, and CI represent Al-induced secretion of malate, oxalate, and citrate from roots, respectively. Differences between the two Al levels were analyzed by ANOVA. Different letters in each row indicate significant differences (Duncan’s test; P ≤ 0.05).
