# Supplementary material for: Phosphorus application reduces aluminum toxicity in two Eucalyptus clones by increasing its accumulation in roots and decreasing its content in leaves
Source: PLoS One. 2018 Jan 11;13(1):e0190900. doi: 10.1371/journal.pone.0190900 (PMC5764327; doi:10.1371/journal.pone.0190900)
Supplement: S5 Table — Note: Differences between the two clones were analyzed by ANOVA. Different letters in each row indicate significant differences (Duncan’s test; P ≤ 0.05). (DOCX) [file pone.0190900.s005.docx]

S5 Table. Duncan’s multiple range test in different clones for Al-induced secretion of organic acids from roots

| Clone | MA | OX | CI |
| --- | --- | --- | --- |
| DH 32-29 | 4.10 ± 0.95 b | 2.86 ± 0.69 b | 0.22 ± 0.06 b |
| G9 | 4.96 ± 1.11 a | 3.74 ± 0.53 a | 0.38 ± 0.09 a |

Note: Differences between the two clones were analyzed by ANOVA. Different letters in each row indicate significant differences (Duncan’s test; P ≤ 0.05).
