# Supplementary material for: Phosphorus application reduces aluminum toxicity in two Eucalyptus clones by increasing its accumulation in roots and decreasing its content in leaves
Source: PLoS One. 2018 Jan 11;13(1):e0190900. doi: 10.1371/journal.pone.0190900 (PMC5764327; doi:10.1371/journal.pone.0190900)
Supplement: S6 Table — Note: The abbreviations PE, ME, MD, CS, ID, and AC represent the activities of PEPC, NADP-ME, NAD-MDH, CS, NAD-IDH, and Cyt-ACO in roots, respectively. Differences between the two Al levels were analyzed by ANOVA. Different letters in each row indicate significant differences (Duncan’s test; P ≤ 0.05). (DOCX) [file pone.0190900.s006.docx]

S6 Table. Duncan’s multiple range test with or without Al stress for enzyme activities in roots

| Al (mM) | PE | ME | MD | CS | ID | AC |
| --- | --- | --- | --- | --- | --- | --- |
| 0 | 416.22 ± 97.22 b | 26.27 ± 11.37 b | 52.75 ± 3.56 b | 218.94 ± 44.84 b | 61.17 ± 11.36 a | 119.16 ± 30.19 a |
| 5 | 563.37 ± 93.87 a | 39.30 ± 14.83 a | 91.82 ± 36.37 a | 261.65 ± 60.06 a | 26.10 ± 13.57 b | 37.11 ± 23.84 b |

Note: The abbreviations PE, ME, MD, CS, ID, and AC represent the activities of PEPC, NADP-ME, NAD-MDH, CS, NAD-IDH, and Cyt-ACO in roots, respectively. Differences between the two Al levels were analyzed by ANOVA. Different letters in each row indicate significant differences (Duncan’s test; P ≤ 0.05).
