# Supplementary material for: Phosphorus application reduces aluminum toxicity in two Eucalyptus clones by increasing its accumulation in roots and decreasing its content in leaves
Source: PLoS One. 2018 Jan 11;13(1):e0190900. doi: 10.1371/journal.pone.0190900 (PMC5764327; doi:10.1371/journal.pone.0190900)
Supplement: S7 Table — Note: Differences between the two clones were analyzed by ANOVA. Different letters in each row indicate significant differences (Duncan’s test; P ≤ 0.05). (DOCX) [file pone.0190900.s007.docx]

S7 Table. Duncan’s multiple range test in different clones for enzyme activities in roots

| Clone | PE | ME | MD | CS | ID | AC |
| --- | --- | --- | --- | --- | --- | --- |
| DH 32-29 | 411.35 ± 100.33 b | 43.21 ±11.46 a | 54.97 ± 4.95 b | 194.91 ± 23.71 b | 49.31 ± 24.40 a | 59.03 ± 33.91 b |
| G9 | 568.23 ± 81.43 a | 22.35 ± 8.80 b | 89.61 ± 38.52 a | 285.68 ± 40.72 a | 37.97 ± 17.34 b | 97.24 ± 55.87 a |

Note: Differences between the two clones were analyzed by ANOVA. Different letters in each row indicate significant differences (Duncan’s test; P ≤ 0.05).
